# Correlation between circulating tumor DNA and carcinoembryonic antigen levels in patients with metastatic colorectal cancer

**DOI:** 10.1002/cam4.4384

**Published:** 2021-11-24

**Authors:** Hiroki Osumi, Eiji Shinozaki, Akira Ooki, Keitaro Shimozaki, Daisaku Kamiimabeppu, Izuma Nakayama, Takeru Wakatsuki, Mariko Ogura, Daisuke Takahari, Keisho Chin, Kensei Yamaguchi

**Affiliations:** ^1^ Department of Gastroenterology Cancer Institute Hospital, Japanese Foundation for Cancer Research Tokyo Japan

**Keywords:** carcinoembryonic antigen, circulating tumor DNA, liquid biopsy, metastatic colorectal cancer, metastatic organ

## Abstract

**Background:**

Circulating tumor DNA (ctDNA) is a biomarker with potential to reflect comprehensive genomic information and overcome intratumor heterogeneity. In contrast, carcinoembryonic antigen (CEA) is a conventional tumor marker for predicting recurrence, survival, and chemotherapeutic efficacy in patients with metastatic colorectal cancer (mCRC). However, the relationship between them remains unclear. Here, the relationship between plasma ctDNA and CEA levels was evaluated to clarify the advantages and disadvantages of their clinical use.

**Methods:**

A total of 110 patients with mCRC underwent chemotherapy were enrolled. Amplicon‐based plasma genomic profiling of 14 genes that are commonly mutated in CRC by next‐generation sequencing was compared to the CEA level and tumor diameter using Spearman’s correlation coefficient.

**Results:**

The overall concordance rate between the ctDNA and CEA levels was 75.5% (83/110). The correlation coefficient between the ctDNA and CEA levels was lower in the group of patients without liver and lymph node metastases (*r* = 0.18, *p* = 0.44) than in the group of patients with liver metastasis (*r* = 0.48, *p* < 0.0001). Although the correlation coefficients between tumor diameter and both ctDNA and CEA levels were high in the group of patients with liver metastasis, only the CEA correlation coefficient was maintained in the group of patients without liver and lymph node metastases (*r* = 0.53, *p* = 0.01). The characteristics that influenced discordance were liver metastasis and the sum of tumor diameter.

**Conclusions:**

The status of ctDNA and CEA may not be consistent in patients with mCRC without liver metastasis or with a low tumor volume; both results should be considered when deciding a treatment strategy.

## Graphical abstract

Tumor diameter and ctDNA and CEA levels highly correlated in metastatic colorectal cancer patients with liver metastases. Status of ctDNA and CEA may not be consistent in metastatic colorectal cancer (mCRC) patients without liver metastasis or with low tumor volume. Both results should be considered when deciding a treatment strategy for mCRC patients without liver metastases.

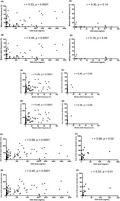

## 1 INTRODUCTION

Currently, colorectal cancer (CRC) is one of the most prevalent cancers globally,^1^ with an estimated 18.1 million new cancer cases and 9.6 million cancer‐related deaths.^1^ The number of CRC‐related deaths in Japan have also been increasing, exceeding 50,000.^2^ Although the prognosis for patients with metastatic CRC (mCRC) is especially poor, it has improved because of improvements in diagnosis and advancements in multidisciplinary treatment.^3^ Among the diagnostic methods, liquid biopsy has garnered attention as a minimally invasive method that enables the characterization of the cancer genome of patients with cancer.^4^, ^5^ Liquid biopsy was reported several years ago as a blood test that provides the same genomic information contained as a tissue biopsy.^6^, ^7^ It includes the analysis of circulating tumor DNA (ctDNA), circulating tumor cells, and exosomes secreted from cancer cells in body fluids, such as peripheral blood, urine, and cerebrospinal fluid.^4^, ^5^ Of these, ctDNA is one of the most extensively studied technologies because the recent development of highly sensitive next‐generation sequencers has enabled comprehensive characterization of cancer cells and detection of the changes in tumor genotype.^4^, ^5^ Recently, the Food and Drug Administration approved Guardant360^®^ CDx and FoundationOne^®^ Liquid CDx for tumor mutation profiling, which may be used for comprehensive genomic profiling of patients with any solid malignant neoplasm.^8^, ^9^ Thus, ctDNA monitoring may be a useful biomarker for tumor recurrence, drug resistance, and treatment efficacy, which enables physicians to choose more appropriate treatments for each patient.^10^ However, the challenges associated with using ctDNA include the accumulation of clinical evidence and dependence of results on tumor volume and the type of metastatic organ.^11^ Thus, ctDNA may not be detectable, even in patients with mCRC.

However, conventional serum tumor markers, such as carcinoembryonic antigen (CEA), are widely used in clinical practice. Gold et al. first reported CEA in 1965 as a common fetal antigen for CRC.^12^ CEA is a well‐established, low‐cost biological tumor marker for CRC.^13^ It is widely used in CRC as a follow‐up for postoperative recurrence and as a predictor of chemotherapy response, and is recommended by several clinical treatment guidelines for CRC.^13^, ^14^ Similar to ctDNA—as CEA is not specific to CRC—the CEA test may not be positive in patients with mCRC. Moreover, the test is positive in patients with various cancers and benign disorders and individuals who smoke.

Several studies have compared tissue biopsy and liquid biopsy,^11^ but only a few have compared and correlated CEA and ctDNA levels in detail.^15^ In our previous study, we revealed that the ctDNA level (mutant allele frequency) was significantly associated with liver and lymph node metastasis, tumor markers (CEA and CA19‐9) and tumor diameter. However, it remains unclear that the association of ctDNA level with CEA level changed, depending on the metastatic organ and whether ctDNA or CEA is associated with tumor diameter more accurately.^16^ As both would be used in routine clinical practice in the near future, understanding the relationship between the ctDNA and CEA levels is useful for optimizing treatment change and selection because it can predict the efficacy of chemotherapy for mCRC. In this study, the relationship between plasma ctDNA and CEA levels was evaluated to clarify the advantages and disadvantages in clinical use.

## 2 MATERIALS AND METHODS

### 2.1 Patients

The primary endpoint was to evaluate the correlation between ctDNA and CEA levels in patients with mCRC. One hundred and ten patients with mCRC who received chemotherapy in the Cancer Institute Hospital, Japanese Foundation for Cancer Research, were consecutively enrolled in this study between February 2017 and March 2018. In this study, we evaluated the aforementioned aspects, using the subjects of our previous two studies.^16^, ^17^ A specific course of treatment and specific time points for collecting blood samples were not required for the enrollment of subjects in this study because we used data including the feasibility study of ctDNA analysis for detecting prevalent mutations in CRC (Table 1).^16^, ^17^ Blood collection for ctDNA and CEA was done at the same time. This study was approved by the Institutional Review Board of the Japanese Foundation for Cancer Research (Tokyo, Japan). Written informed consent was obtained from all patients.

**TABLE 1:** Patient demographics and clinical characteristics

Characteristic	Total (*N* = 110) No. of patients (%)
Age at enrollment, years	
Median [range]	62.0 [30.0–84.0]
Sex	
Male	66 (60.0)
Female	44 (40.0)
Treatment line at the time of sampling	
Neoadjuvant chemotherapy	12 (10.9)
First line	37 (33.6)
Second line	36 (32.7)
Third or later line	17 (15.5)
Adjuvant chemotherapy	8 (7.3)
Treatment at registration at the time of sampling	
FOLFIRI/CPT−11+anti‐VEGF antibody	47 (42.7)
SOX/CapeOX/FOLFOX/FOLFOXIRI+anti‐VEGF antibody	18 (16.4)
FOLFOX+anti‐EGFR antibody	15 (13.6)
FOLFIRI/CPT−11+anti‐EGFR antibody	10 (9.1)
FOLFOX	5 (4.5)
Regorafenib	4 (3.6)
CapeOX	3 (2.7)
TAS102	3 (2.7)
5‐FU+LV/Capecitabine+anti‐VEGF antibody	2 (1.8)
TAS102+anti‐EGFR antibody	2 (1.8)
Capecitabine	1 (0.9)
Primary site	
Right‐side colon	28 (25.5)
Left‐side colon	82 (74.5)
Resection of primary tumor	
Yes	75 (68.2)
No	35 (31.8)
Metastatic site	
Single organ	43 (39.1)
Multi organ	67 (60.9)
Liver	84 (76.4)
Lung	44 (44.0)
Lymph node	34 (34.9)
Peritoneum	25 (22.7)
Others	20 (18.2)
*RAS* status in tissue	
Wild type	62 (56.4)
Mutant	48 (43.6)
Tumor markers	
CEA median, [range]	16.0 [1–7479]
CA19‐9 median, [range]	28.1 [2–≥50,000]

Abbreviations: 5‐FU, fluorouracil; CA19‐9, carbohydrate antigen 19–9; CapeOX, a combination of capecitabine with oxaliplatin; CEA, carcinoembryonic antigen; CPT‐11, irinotecan hydrochloride hydrate; EGFR, epidermal growth factor receptor; FOLFIRI, a combination of calcium folinate and fluorouracil with irinotecan hydrochloride hydrate; FOLFOX, a combination of calcium folinate and fluorouracil with oxaliplatin; FOLFOXIRI, a combination of calcium folinate and fluorouracil, and irinotecan hydrochloride hydrate with oxaliplatin; LV, calcium folinate; *RAS*, rat sarcoma viral oncogene homolog; SOX, a combination of tegafur, gimeracil, and oteracil potassium with oxaliplatin; TAS102, trifluridine, tipiracil hydrochloride; VEGF, vascular endothelial growth factor.

### 2.2 Blood sampling, circulating tumor DNA isolation, and sequencing

Blood samples were collected in tubes containing ethylenediaminetetraacetic acid following the manufacturer’s instructions.^16^ Plasma was obtained by centrifuging the blood samples at 1600 *g* for 10 min at 4°C, and then at 16,000 *g* for 10 min at 4°C to remove cellular debris.^16^ Circulating cell‐free DNA (cfDNA) was extracted from 2 ml of plasma using a MagMAX cell‐free DNA Isolation Kit (Thermo Fisher Scientific), following the manufacturer's instructions.^16^ The Oncomine Colon cfDNA Assay (Thermo Fisher Scientific) was used to generate libraries from cfDNA following the manufacturer's instructions.^16^ Quality control of the libraries was performed using Qubit^®^ 2.0 and 2100 Bioanalyzer (Agilent Technologies).^16^ The Ion Chef™ System and Ion 530™ Kit‐Chef (Thermo Fisher Scientific) were used for template preparation, followed by sequencing on an Ion S5 system using Ion 530 chips.^16^ A six‐plex library pool was applied to the Ion 530 chips.^16^ The sequencing data of the quality control passing samples were then uploaded into the Ion Reporter^TM^ Analysis Server for variant calling and annotation according to the manufacture instruction.

The cfDNA panel used in this study included 14 genes with >240 hotspots (single‐nucleotide variants and short indels), namely, AKT serine/threonine kinase 1 (*AKT1*), Raf murine sarcoma viral oncogene homolog B (*BRAF*), catenin beta 1 (*CTNNB1*), epidermal growth factor receptor (*EGFR*), Erb‐B2 receptor tyrosine kinase 2 (*ERBB2*), F‐box and WD repeat domain containing 7 (*FBXW7*), guanine nucleotide binding protein, alpha stimulating (*GNAS*), Kirsten rat sarcoma viral oncogene homolog (*KRAS*), mitogen‐activated protein kinase kinase 1 (*MAP2K1*), neuroblastoma RAS viral oncogene homolog (*NRAS*), phosphatidylinositol‐4,5‐bisphosphate 3‐kinase catalytic subunit alpha (*PIK3CA*), mothers against decapentaplegic homolog 4 (*SMAD4*), p53 (*TP53*), and adenomatous polyposis coli (*APC)*.^16^ Clean reads were mapped to the human reference genome (hg19).^16^ Variant Caller was used to filter and call the mutations in the targeted regions of each gene.^18^, ^19^ A median of 20 million reads were generated per spike‐in sample 530 chip. The average coverage ranged from 30,000 to 110,000 and the average molecular coverage ranged from 1000 to 8000. The cut‐off level for each mutant allele frequency was defined by “variant caller” for each sample (patient). We used the following filtering parameters for variant calling: (i) the minimum number of variant allele reads were ≥10, (ii) the coverage depth was ≥20, (iii) Common single nucleotide polymorphisms = Not In, and (iv) Confident Somatic Variants = In.^20^ In this study, the cut‐off value for the mutant allele fraction (MAF) was 0.15%.

### 2.3 Statistical analysis

The association among ctDNA—which was the highest allele frequency of the detected mutant alleles in each patient—CEA level, and tumor diameter was investigated using the Spearman’s rank correlation coefficient. There were four groups according to the metastatic organ: (i) all patients (*n* = 110), (ii) group that included patients with liver metastasis (*n* = 84, lung: 30, lymph node: 29, peritoneum: 20, others: 19), (iii) group that did not include patients with liver metastasis; (*n* = 26, lung: 14, lymph node: 5, peritoneum: 5, others: 9), and (iv) group that did not include patients with liver and lymph node metastases (*n* = 21, lung: 11, peritoneum: 4, others: 9). CEA positive was defined as a CEA level ≥5 ng/ml and ctDNA positive as an MAF ≥0.15. Patients concordantly positive or negative for both CEA and ctDNA were included in the CEA/ctDNA concordance group. The differences in clinical factors between the ctDNA/CEA status concordance group and the ctDNA/CEA status discordance group were evaluated using Welch’s *t*‐test. Statistical tests used two‐sided *p* values with a significance level of <0.05. All statistical analyses were performed using the statistical software GraphPad Prism 7 (Prism 7 Resourcing Ltd.).

## 3 RESULTS

### 3.1 Patient characteristics

One hundred and ten patients with mCRC who received chemotherapy in neoadjuvant, adjuvant, or metastatic settings were recruited. The characteristics of the 110 patients are summarized in Table 1. The median age of patients at the time of recruitment was 62.0 (range, 30.0–84.0) years, and 66 were men (60.0%). The liver was the most frequent site of metastasis (76.4%), followed by the lungs (44.0%), lymph nodes (34.9%), and peritoneum (22.7%). Of the 110 patients, 62 (56.4%) showed wild‐type *RAS* in their tissues obtained from tissue biopsies or surgical specimens (Table 1).

### 3.2 Detection of somatic mutations in plasma

Of the 110 patients recruited, one or more somatic mutations in the 14 CRC‐related genes were detected in 78 (70.9%) plasma samples of patients with mCRC. Mutations in *TP53*, *KRAS*, and *APC* were detected in 72 (65.5%), 42 (38.2%), and 26 (23.6%) patients, respectively. *FBXW7* and *PIK3CA* were also frequently mutated in 17 (15.5%) and 15 (13.6%) patients, respectively. Mutations in *GNAS* (8.2%), *BRAF* (7.3%), *SMAD4* (5.5%), *NRAS* (3.6%), *MAP2K1* (3.6%), *EGFR* (3.6%), *ERBB2* (1.8%), and *CTNNB1* (0.9%) were less common (<10% of patients) than those in the above genes. In this study, only RAS mutation was examined using tissue specimen. The overall concordance rate of *RAS* status between tissue and ctDNA in 110 patients was 74.5% (82/110).

### 3.3 Association of the ctDNA level with the CEA level in patients with mCRC

**FIGURE 1:**
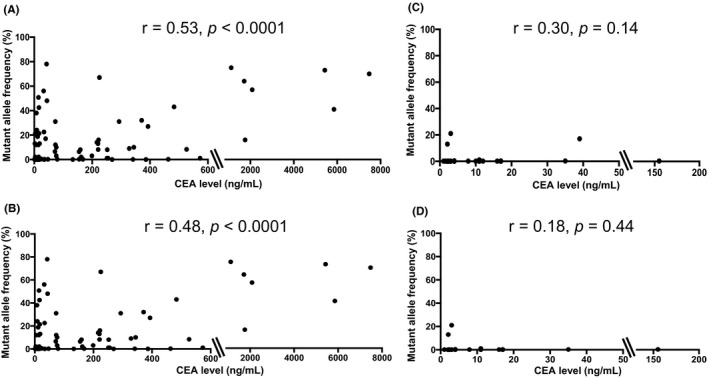
Correlation between the circulating tumor DNA (ctDNA) and carcinoembryonic antigen (CEA) levels. (A) All patients (*n* = 110), (B) group that included patients with liver metastasis (*n* = 84), (C) group that did not include patients with liver metastasis (*n* = 26), and (D) group that did not include patients with liver and lymph node metastases (*n* = 21)

**FIGURE 2:**
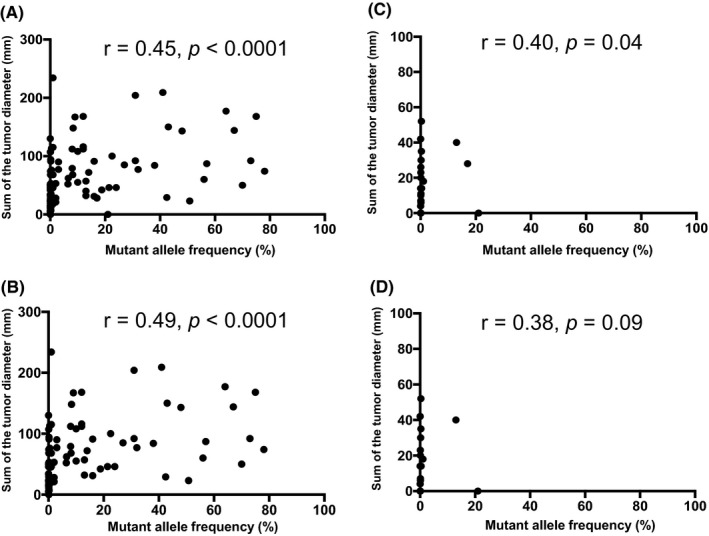
Correlation between the circulating tumor DNA (ctDNA) level and the sum of the longest tumor diameters by response evaluation criteria in solid tumors (RECIST). (A) All patients (*n* = 110), (B) group that included patients with liver metastasis (*n* = 84), (C) group that did not include patients with liver metastasis (*n* = 26), and (D) group that did not include patients with liver and lymph node metastases (*n* = 21)

**FIGURE 3:**
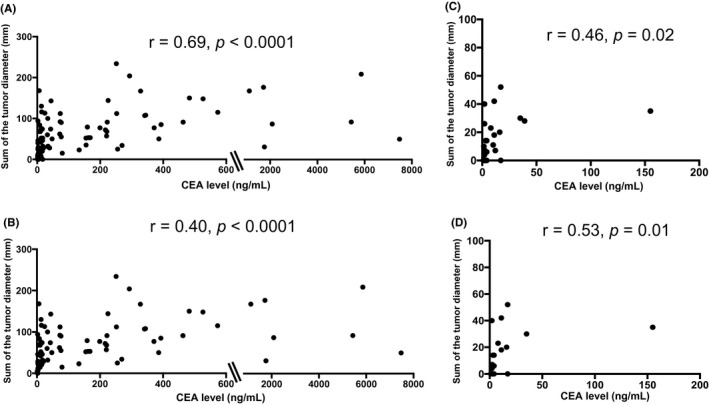
Correlation between the carcinoembryonic antigen (CEA) level and the sum of longest tumor diameters by response evaluation criteria in solid tumors (RECIST). (A) All patients (*n* = 110), (B) group that included patients with liver metastasis (*n* = 84), (C) group that did not include patients with liver metastasis (*n* = 26), and (D) group that did not include patients with liver and lymph node metastases (*n* = 21)

To assess the clinical utility and difference between the ctDNA and CEA levels in patients with mCRC, both associations between the ctDNA and CEA levels and the sum of tumor diameters were investigated, as estimated from standard response evaluation criteria in solid tumors (RECIST) in patients with mCRC. The median ctDNA level was 1.0 (range 0.15–78.6). The CEA level was elevated at baseline in 83 of the 110 patients (75.5%), with a median baseline level of 16.0 (range: 1–7479). The correlation coefficient between the ctDNA and CEA levels was 0.53 (*p* < 0.0001) in all patients (Figure 1). In contrast, the correlation coefficients were lower in the group that did not include patients with liver and lymph node metastases (*r* = 0.18, *p* = 0.44) than in the group that included patients with liver metastasis (*r* = 0.48, *p *< 0.0001) (Figure 1). The correlation coefficients between the ctDNA/CEA level and tumor diameter were similar in all patients and the group that included patients with liver metastasis (all patients: ctDNA: *r* = 0.45, *p* < 0.0001; CEA: *r* = 0.69, *p* < 0.0001; liver metastasis: ctDNA: *r* = 0.49, *p* < 0.0001, CEA: *r* = 0.40, *p* < 0.0001) (Figures 2 and 3). However, only the correlation coefficient between the CEA level and tumor diameter was maintained, even in the group that did not include patients with liver and lymph node metastases (*r* = 0.53, *p* = 0.01) (Figures 2 and 3).

### 3.4 Clinical features of discordant cases between the ctDNA and CEA levels

To investigate the clinical features of discordant cases between the ctDNA and CEA levels, the clinical features between patients with discordant results and those with concordant results (both positive cases) were compared. The sensitivity of ctDNA, CEA, and combination of both ctDNA and CEA were 70.9% (78/110), 75.5% (83/110), and 85.5% (94/110), respectively. In the group that included patients with liver metastasis (*n *= 84), the sensitivity of ctDNA, CEA, and combination of both ctDNA and CEA were 82.1% (69/84), 85.7% (72/84), and 94.0% (79/84), respectively. On the other hand, in the group that included patients without liver metastasis (*n *= 26), the sensitivity of ctDNA, CEA and combination of both ctDNA and CEA were 34.6% (9/26), 42.3% (11/26) and 57.7% (15/26), respectively. Furthermore, in the group that included patients with lymph node metastasis (*n *= 34), the sensitivity of ctDNA, CEA and combination of both ctDNA and CEA were 73.5% (25/34), 76.5% (26/34) and 85.3% (29/34), respectively. On the other hand, in the group that included patients without lymph node metastasis (*n *= 76), the sensitivity of ctDNA, CEA, and combination of both ctDNA and CEA were 69.7% (53/76), 75.0% (57/76), and 85.5% (65/76), respectively.

The overall concordance rate between the ctDNA and CEA levels was 75.5% (83/110) (Table 2), indicating that 27 patients had a discordant status between the ctDNA and CEA levels. We found that 50% of the ctDNA‐negative cases were CEA positive (16/32). As shown in Table 3, the ctDNA level in the discordant cases was significantly lower than that in the concordant cases (ctDNA levels of 0.34% and 9.0%, respectively; *p* = 0.005). Furthermore, the CEA level in the discordant cases was significantly lower than that in the concordant cases (CEA level 13.1 ng/ml and 73.0 ng/ml, respectively; *p* = 0.027, Table 3). The cause of the discordant status between the ctDNA and CEA levels was further investigated (Table 3). The clinical features between the discordant cases (ctDNA positive/CEA negative: *n* = 11 or ctDNA negative/CEA positive: *n* = 16) and concordant cases (both positive: *n* = 67) were compared. There were significant differences in the frequency of liver metastasis, resection of the primary tumor, and sum of the tumor diameters between the ctDNA‐positive/CEA‐negative cases and concordant cases (liver metastasis, *p* = 0.02; resection of primary tumor, *p* = 0.006; tumor diameter, *p* < 0.0001; Table 3). Similarly, there were significant differences in the sex ratio and frequency of liver metastasis, and tumor diameter between the ctDNA‐negative/CEA‐positive cases and concordant cases (sex: *p* = 0.048, liver metastasis: *p* = 0.005, tumor diameter: *p* < 0.0001, Table 3). The clinical features of patients with discordant and concordant results (both negative cases) are also summarized in Table 3.

**TABLE 2:** Relationship between ctDNA and CEA levels

		CEA		Total
		Negative	Positive	
ctDNA	Negative	16	16	32
	Positive	11	67	78
	Total	27	83	110

Abbreviations: CEA, carcinoembryonic antigen; ctDNA, circulating tumor DNA.

**TABLE 3:** Comparison of patient demographics and clinical characteristics according to the ctDNA and CEA status

Characteristics	ctDNA positive CEA positive (*N* = 67)	CEA negative ctDNA positive (*N* = 11)	*p* value*	ctDNA negative CEA positive (*N* = 16)	*p* value**	ctDNA negative CEA negative (*N* = 16)	*p* value***
Age at enrollment, years							
Median [range]	64.0 [32–82]	69.0 [36–80]	0.66	62.0 [30–74]	0.66	56.5 [30–73]	0.028
Sex							
Male	35 (52.2)	9 (81.8)	0.1	13 (81.2)	0.048	9 (56.2)	0.1
Female	32 (47.8)	2 (18.2)		3 (18.8)		7 (43.8)	
Primary site							
Right‐sided colon	21 (31.0)	5 (19.2)	0.31	3 (18.8)	0.37	2 (12.5)	0.5
Left‐sided colon	47 (69.0)	21 (80.8)		13 (81.2)		14 (87.5)	
Metastatic site							
Single organ	19 (28.4)	4 (36.4)	0.36	4 (36.4)	0.36	11 (68.8)	0.004
Multi organ	48 (71.6)	7 (63.6)		7 (63.6)		5 (31.2)	
Liver	62 (92.5)	7 (63.6)	0.02	10 (62.5)	0.005	5 (31.2)	<0.0001
Lung	28 (41.8)	5 (45.5)	1	5 (31.2)	1	6 (37.5)	1
Peritoneal	20 (29.9)	2 (18.2)	0.72	2 (18.2)	0.72	1 (6.2)	0.06
Lymph node	22 (32.8)	3 (27.3)	1	2 (12.5)	1	5 (31.2)	1
Resection of primary tumor							
Yes	39 (58.2)	11 (100)	0.006	11 (68.8)	0.57	14 (87.5)	0.04
No	28 (41.8)	0 (0)		5 (31.2)		2 (12.5)	
*RAS* status in tissue							
Wild‐type	32 (47.8)	6 (54.5)	0.72	11 (68.2)	0.17	12 (75.0)	0.057
Mutant	35 (52.2)	5 (45.5)		5 (31.2)		4 (25.0)	
Sum of the tumor diameter							
Median [range]	68 [15.0–234.0]	14 [1.0–94.0]	<0.0001	27.5 [1.0–130.0]	<0.0001	6.0 [1.0–46.0]	<0.0001
Maximum ctDNA level							
Median [range]	9.0 [0.15–78.1]	0.34 [0.15–21.0]	0.005	0.07 [0.00–0.14]	<0.0001	0.08 [0.0–0.13]	<0.0001
CEA level							
Median [range]	73.0 [6.0–7479]	3.0 [2.0–4.0]	<0.0001	13.1 [5.0–386.0]	0.027	2.4 [1.0–4.0]	<0.0001

Abbreviations: CEA, carcinoembryonic antigen; ctDNA, circulating tumor DNA; NA, not applicable; *RAS,* rat sarcoma viral oncogene homolog.

* *p* value for the comparison between the ctDNA positive CEA‐positive group and the CEA negative ctDNA‐positive group.

** *p* value for the comparison between the ctDNA positive CEA‐positive group and the ctDNA negative CEA‐positive group.

*** *p* value for the comparison between the ctDNA positive CEA‐positive group and the ctDNA negative CEA‐negative group.

## 4 DISCUSSION

In this study, the correlation between the ctDNA and CEA levels in patients with mCRC was evaluated. The correlation between the ctDNA and CEA levels tended to be low in mCRC patients with small tumor diameter and without liver metastasis. Furthermore, both ctDNA and CEA levels were affected by tumor volume, and the incidence of false negatives increased in smaller tumor cases. However, only the correlation coefficient between the CEA level and tumor diameter was maintained, even in the group that did not include patients with liver and lymph node metastases. Generally, the half‐life of CEA (a few days to weeks) is longer than that of cfDNA in circulation (minutes to several hours).^21^, ^22^ Another possibility that may cause the above discordant status between the ctDNA and CEA levels is the intrinsic biological characteristics between metastatic sites.^23^, ^24^, ^25^ A significant association was observed between the ctDNA level and liver metastasis, whereas no association was observed in patients without liver metastasis. Similarly, Vidal et al. reported that the site of peritoneal and lung metastases and the mucinous histology of the tumor negatively affect the detection of ctDNA.^23^ Furthermore, Bando et al. reported that only lung metastasis is the most important factor associated with the results of tissue–plasma discordance.^24^ These results suggest that there is a high correlation between the ctDNA level and liver metastasis and a low correlation between the ctDNA level and lung or peritoneal metastasis. One of the possible causes of the above discordance among metastatic sites is the difference in the distribution of DNAase, depending on the metastatic site.^26^ In the lung, the clearance of ctDNA may be higher because of a higher level of DNAase, resulting in a lower ctDNA level.^26^ This is supported by the finding of a previous study, which reported that the detection rate of the T790M mutation in intrapulmonary metastasis is lower than that of extrapulmonary metastasis in lung cancer.^27^ Therefore, in the case of metastasis that did not include liver metastasis, especially lung metastasis, it may be necessary to evaluate the combination of existing tumor markers because the ctDNA false‐negative rate may be high.

This study had some limitations. First, because there were only a few patients with a single site of metastases, it was difficult to discuss only lung or peritoneal metastasis. In addition, there might be mutations in genes or alleles, other than the panel of 14 genes evaluated in this study. Second, the frequencies of mutated genes in plasma ctDNA did not match the frequency in tissue DNA reported in mutation databases such as The Cancer Genome Atlas.^28^ Although the mutation frequency of *APC* in CRC has been reported to be around 80%,^28^ the results of this study revealed a mutation frequency of only 23.6%. This difference can be partially explained by the insufficient coverage of the panel used in this study for detecting *APC* mutations.^16^ To promote the utility and optimize the routine evaluation of ctDNA determination in daily clinical practice, further external factors that may influence the results of ctDNA determination should be studied.

In conclusion, our findings confirmed that the status of ctDNA and CEA might not be consistent in patients with mCRC but without liver metastasis or with low tumor volume, and both results should be taken into account when deciding a treatment strategy.

## CONFLICT OF INTEREST

The authors declare that they have no conflict of interest in this study.

## CONSENT FOR PUBLICATION

Written informed consent was obtained from all patients.

## ETHICS APPROVAL AND CONSENT TO PARTICIPATE

All procedures followed were in accordance with the ethical standards of the responsible committee on human experimentation (The Cancer Institute Hospital of Japanese Foundation for Cancer Research, Institutional Review Board, approval number 2017–1009) and with the Helsinki Declaration.

## Data Availability

The data that support the findings of this study are available from the corresponding author upon reasonable request.
